# Influence of genomic variations on glanders serodiagnostic antigens using integrative genomic and transcriptomic approaches

**DOI:** 10.3389/fvets.2023.1217135

**Published:** 2023-12-06

**Authors:** Philippe Charron, Ruimin Gao, John Chmara, Emily Hoover, Susan Nadin-Davis, Danielle Chauvin, Jennifer Hazelwood, Kennedy Makondo, Marc-Olivier Duceppe, Mingsong Kang

**Affiliations:** Ottawa Laboratory-Fallowfield, Canadian Food Inspection Agency, Ottawa, ON, Canada

**Keywords:** *Burkholderia mallei*, genomic variation, serodiagnostic antigen, transcriptome, virulence factors

## Abstract

Glanders is a highly contagious and life-threatening zoonotic disease caused by *Burkholderia mallei* (*B. mallei*). Without an effective vaccine or treatment, early diagnosis has been regarded as the most effective method to prevent glanders transmission. Currently, the diagnosis of glanders is heavily reliant on serological tests. However, given that markedly different host immune responses can be elicited by genetically different strains of the same bacterial species, infection by *B. mallei*, whose genome is unstable and plastic, may result in various immune responses. This variability can make the serodiagnosis of glanders challenging. Therefore, there is a need for a comprehensive understanding and assessment of how *B. mallei* genomic variations impact the appropriateness of specific target antigens for glanders serodiagnosis. In this study, we investigated how genomic variations in the *B. mallei* genome affect gene content (gene presence/absence) and expression, with a special focus on antigens used or potentially used in serodiagnosis. In all the genome sequences of *B. mallei* isolates available in NCBI’s RefSeq database (accessed in July 2023) and in-house sequenced samples, extensive small and large variations were observed when compared to the type strain ATCC 23344. Further pan-genome analysis of those assemblies revealed variations of gene content among all available genomes of *B. mallei*. Specifically, differences in gene content ranging from 31 to 715 genes with an average of 334 gene presence-absence variations were found in strains with complete or chromosome-level genome assemblies, using the ATCC 23344 strain as a reference. The affected genes included some encoded proteins used as serodiagnostic antigens, which were lost due mainly to structural variations. Additionally, a transcriptomic analysis was performed using the type strain ATCC 23344 and strain Zagreb which has been widely utilized to produce glanders antigens. In total, 388 significant differentially expressed genes were identified between these two strains, including genes related to bacterial pathogenesis and virulence, some of which were associated with genomic variations, particularly structural variations. To our knowledge, this is the first comprehensive study to uncover the impacts of genetic variations of *B. mallei* on its gene content and expression. These differences would have significant impacts on host innate and adaptive immunity, including antibody production, during infection. This study provides novel insights into *B. mallei* genetic variants, knowledge which will help to improve glanders serodiagnosis.

## Introduction

Glanders is an infectious and usually fatal zoonosis caused by *Burkholderia mallei* (*B. mallei*). Compared to other species in the family Burkholderiaceae, most of which are soil residents, *B. mallei* is a mammalian intracellular pathogen (1). Horses are highly susceptible to *B. mallei* infections and have been regarded as the natural reservoir (2). Other solipeds, such as mules and donkeys, are also susceptible to *B. mallei* infection, as well as humans (2). As *B. mallei* is highly infectious through aerosols, it was used as a biological weapon during World War I and has since been classified as a category B bioterrorism agent by the Centers for Disease Control and Prevention. Glanders has now been eradicated from most developed countries, however, it remains endemic in a number of Asian, European, African, Middle Eastern, and South American countries where sporadic infections have been detected, including Germany, Russia, Brazil, India, Turkey, and China, and significantly affects regional economic activities and international trade (3, 4). These incidents highlight the potential for importing the glanders agent to glanders-free countries or areas, thus posing a significant risk to equine and public health with huge economic loss and potentially fatal consequences globally.

So far, due to the absence of licensed vaccines or preventive treatment against glanders (5), a fast and accurate early diagnosis remains the most efficient way to prevent *B. mallei* transmission and infection. Diagnosis of glanders is mainly conducted using serodiagnostic assays for surveillance and international trade of Equidae. The complement fixation test (CFT), a serological test officially recommended by the World Organization for Animal Health (WOAH, founded as OIE) with decades of utilization in the equine industry, represents a practical method and is currently the internationally accepted approach for glanders diagnosis. However, the specificity of CFT may vary and its standardization has been questioned for years (4, 6). In order to improve the accuracy of glanders diagnosis, the immunoblot (IB) assay using Lipopolysaccharides (LPS) antigen (7) has been used to confirm CFT “positive” results. However, such a subjective test is still quite technically cumbersome, especially the production of the LPS antigen. In addition, IB may also generate false-positive results due to similar LPS structures between *Burkholderia* species, especially those acting as opportunistic pathogens (8, 9). Therefore, it would be beneficial to establish a simpler, more objective, and quantifiable serological test, such as an ELISA. ELISAs using single or double recombinant protein antigens have been developed and evaluated, and the results are promising (6, 10, 11).

Theoretically, *B. mallei* is believed to have evolved from an ancestral *B. pseudomallei* environmental isolate following an animal infection (12), by the expansion of insertion sequence (IS) elements, prophage elimination, and genome rearrangements, specifically deletions (13). The *B. mallei* genome (5.8 Mb) is 20% smaller than the *B. pseudomallei* genome (7.2 Mb), and many genes needed for environmental survival were lost from *B. mallei*, while those critical for host survival were maintained (13, 14). It has also been shown that the genome of *B. mallei* is significantly less stable than that of *B. pseudomallei*, and large-scale genomic alterations, such as insertions/deletions (INDELs), and structural variants (SVs) related to chromosomal rearrangements, have been observed in many *B. mallei* genomes (15–17). These dynamic processes may impact gene content (gene presence/absence) and expression through various mechanisms (18).

Recently, the WOAH reference laboratory for glanders in France reported that a genetic variant of *B. mallei* compromised the molecular diagnosis of glanders (19). Since interspecies differences in genome sequences, especially the accessory genome, also elicit distinctly different host adaptive immune responses (20), the high plasticity of *B. mallei* genomes may cause more variations in immunological responses that potentially disrupt the reliability of serodiagnosis, a vital tool in detecting glanders. In this study, using integrative genomic and transcriptomic analyses, we characterized the effects of genetic variation on gene content and expression to evaluate currently used and potentially useful serodiagnostic biomarkers to help improve glanders serodiagnosis.

## Materials and methods

### *Burkholderia mallei* genome assemblies from NCBI reference sequence database

All available *Burkholderia mallei* genome assemblies (*n* = 108) and corresponding metadata, including NCBI RefSeq ID, level of assembly, contig count, L50, N50, size, and geographic location (Supplementary File S1), were downloaded from the NCBI Reference Sequence database (as of July 2023) using Bit v1.8.35^1^ and EDirect v15.6.^2^ All *B. mallei* genome assemblies and annotation completeness were evaluated using BUSCO with a completeness score cut-off value of 90% (21–23).

### Bacterial strains and growth condition

*B. mallei* type strain ATCC 23344 was transferred from The National Centre for Foreign Animal Disease (NCFAD) in Winnipeg (24) and strains Zagreb, Mukteswar and Bogor were kindly provided by the Friedrich Loeffler Institute, Federal Research Institute for Animal Health in Germany (7). Live *B. mallei* strains were handled in Containment Level 3 (CL3) facilities. Strains were cultured on a blood agar plate with 3% glycerol for 48 h at 37°C. Cultures were harvested for RNA purification or heat-killed at 85°C for 30 min and the sterility was verified by our standard operating procedure prior to further processing.

### Genomic DNA purification, library preparations and whole genome sequencing using Illumina MiSeq and Nanopore MinION

Genomic DNA was extracted from heat-killed *B. mallei* cultures using the PureLink Genomic DNA Mini Kit (Invitrogen, United States) or NanoBind CBB Kit (PacBio, United States) following the manufacturer’s instructions. The quality and the quantity of extracted DNA were determined by NanoDrop^™^ One^C^ Spectrophotometer and Qubit^™^ 4 Fluorometer, respectively. The extracted genomic DNA samples have a 260/280 ratio of 1.8-2.0 and a 260/230 ratio of 2.0–2.3 with a concentration above 20 ng/μl. Library preparations and whole genome sequencing were performed as described previously (25). Briefly, Illumina (short-read) libraries were prepared using an Illumina DNA Prep (M) Tagmentation kit (Cat.No: 20060059, Illumina, United States), and sequenced on the Illumina MiSeq platform to generate 300-bp paired-end reads. MinION (long-read) libraries were established using Ligation Sequencing Kits (SQK-LSK108 or SQK-LSK109) with Native Barcoding Expansion (EXP-NBD103 or EXP-NBD104) (Oxford Nanopore Technologies, UK) without shearing and were sequenced using an FLO-MIN106 (R9.4.1) flow cell and a MinION Mk1B device.

### Genome assembly and annotation

Whole genome assembly and evaluation were performed as previously described with modifications (25). Briefly, the raw data (FASTQ files) generated from Illumina MiSeq were filtered and trimmed with BBTools v38.87 or Fastp v0.23.2 (26). Raw data (FAST5 files) generated from Nanopore MinION were base-called using Guppy v5.0.11 with the super accuracy mode, trimmed using Porechop v0.2.3,^3^ and filtered using Filtlong v0.2.1.^4^ Hybrid assembly of strain Zagreb using Illumina paired-end and MinION reads was performed by Unicycler v0.4.5 (27). Long read assembly of strains ATCC 23344, Bogor and Mukteswar was performed using Flye v2.9 (28), corrected using Medaka v1.4.4^5^ and polished with Illumina MiSeq reads using a combination of NextPolish v1.4.0 (29) and Polypolish v0.5.0 (30). Nanopore long-read and Illumina short-read data were mapped to the assembled genome sequences using minimap2 (31), and the generated mapping bam files were quality checked. Qualimap (v2.2.1) (32) determined the sequencing coverage depth. The assembled genome was then annotated using the NCBI prokaryotic genome annotation pipeline (PGAP v6.2) (33).

### Genetic variant identification and analysis

Small variants, including SNPs and INDELs, were determined using Snippy v4.6.0 with default parameters,^6^ and SVs were determined using MUM&Co v3.8.0^7^ (34). The core genome SNP alignment obtained from Snippy was used for maximum-likelihood phylogenetic tree construction using IQtree v2.1.2^8^ (35) with 1,000 ultrafast bootstraps. The phylogeny was partitioned into lineages using FastBaps v1.0.8^9^ (36). The phylogenetic tree was visualized using iTOL v6.6.^10^ The distribution of SNPs, INDELs, and SVs was analyzed as described previously (37).

### Pan-genome analysis

Pan-genomes of 112 available *B. mallei* genome sequences were analyzed using PPanGGOLiN v1.2.74^11^ (38), with PGAP–annotated Genbank files as input. In the clustering step, the default parameters were used. Further visualization and analysis of pan-genome results were performed using R v4.2.2, packages ggplot2 v3.4.0,^12^ ggtree v3.2.1 (39), pheatmap v1.0.12,^13^ UpSetR v1.4.0 (40) and vegan v2.6-4.^14^

### RNA isolation and library preparation

Total RNA was extracted from live *B. mallei* strains ATCC 23344 and Zagreb with three biological replicates for each strain in the Containment Level 3 (CL3) laboratory using the PureLink RNA mini kit with the On-column PureLink DNase Treatment (Invitrogen, United States) according to the manufacturer’s instructions. The RNA samples were sent to Centre d’expertise et de service Génome Québec (Québec, Canada) for sequencing. Briefly, total RNA was quantified using a NanoDrop Spectrophotometer ND-1000 (Thermo Scientific, United States), and its integrity was assessed on a 2,100 Bioanalyzer (Agilent Technologies, United States). rRNA were depleted using QIAseq FastSelect -5S/16S/23S kits (QIAGEN, United States). cDNA synthesis was obtained using the NEBNext RNA First Strand Synthesis and NEBNext Ultra Directional RNA Second Strand Synthesis Modules (New England BioLabs, United States). The library preparation was performed using the NEBNext Ultra II DNA Library Prep Kit for Illumina (New England BioLabs, United States), followed by quantification using the Kapa Illumina GA with Revised Primers-SYBR Fast Universal kit (Kapa Biosystems, Switzerland). The libraries were normalized and pooled for sequencing. After ExAMP was added, the pooled library was loaded on an Illumina cBot, and the flowcell was run on a HiSeq 4000 for 2 × 100 cycles (paired-end mode) to generate over 10 million reads for each sample (Supplementary Table S2). A PhiX library was used as a control and mixed with libraries at a 1% level. The Illumina control software was HCS HD 3.4.0.38, and the real-time analysis program was RTA v2.7.7. The bcl2fastq2 v2.20 tool was used to demultiplex samples and generate FASTQ reads.

### Prediction of virulence factors

The file (.faa file) containing coding sequences (CDS) of strain ATCC 23344 was used as an input of PathoFact v1.0 (ORF version) for the prediction of virulence factors (VFs) using default parameters (41). Only CDSs with predicted virulence confidence levels 1 (secreted virulence factor) and 2 (non-secreted virulence factor) were used for further analysis.

### RNA-seq data analysis

Raw reads from the sequenced libraries were quality checked with fastp v0.23.2 (26). The reference cDNA sequences (CDS from genomic FASTA file) of *B. mallei* type strain ATCC 23344 were downloaded from the NCBI RefSeq database. Transcript abundance was quantified using kallisto v0.46.1 (42) with 100 bootstraps. Differential expression analysis was performed using the R package, sleuth v0.30.0 (43). Differential gene expressions (DEGs) were identified based on a fold change >2 and a *q*-value <10^−3^ for further filtering using the Wald test in sleuth. The transcript abundance data were imported into R v4.2.2, and principal coordinates analysis (PCoA) was performed using vegan v2.6-4^15^ and visualized using ggplot2 v3.4.0 (https://cran.r-project.org/web/packages/ggplot2/index.html).

### Kyoto encyclopedia of genes and genomes pathway based on transcriptomic analyzed differentially expressed genes

The identified differentially expressed genes (DEGs) were applied to explore the potentially affected Kyoto encyclopedia of genes and genomes (KEGG) pathways. The corresponding KO (KEGG ORTHOLOGY) table was obtained by GhostKOALA v2.2 (44) using the genus_prokaryotes database. The obtained KO terms corresponding to the DEGs were fed to the KEGG Mapper reconstruction tool (last updated: July 1, 2021)^16^ (45), and the affected pathways were evaluated.

### Effect of genomic variation on gene expression

Based on the simple model that the destruction of operon structure or disruption of the non-coding region upstream of operons can interfere with gene expression (46), variant calls (SNPs, INDELs, and SVs) were compared with RNA-seq data to link DEGs to specific genomic variation. Specifically, we identified the operons using operonSEQer v1.0 (47) and investigated changes in gene expression in relation to an alteration in the operon structure mediated by SVs, as well as changes in the non-coding region upstream of the operon, including promoter and regulators, mediated by SNPs or INDELs. This was achieved using custom scripts modified from a previously described method (48).

## Results

### Genetic variation of *Burkholderia mallei* strains

**Figure 1:**
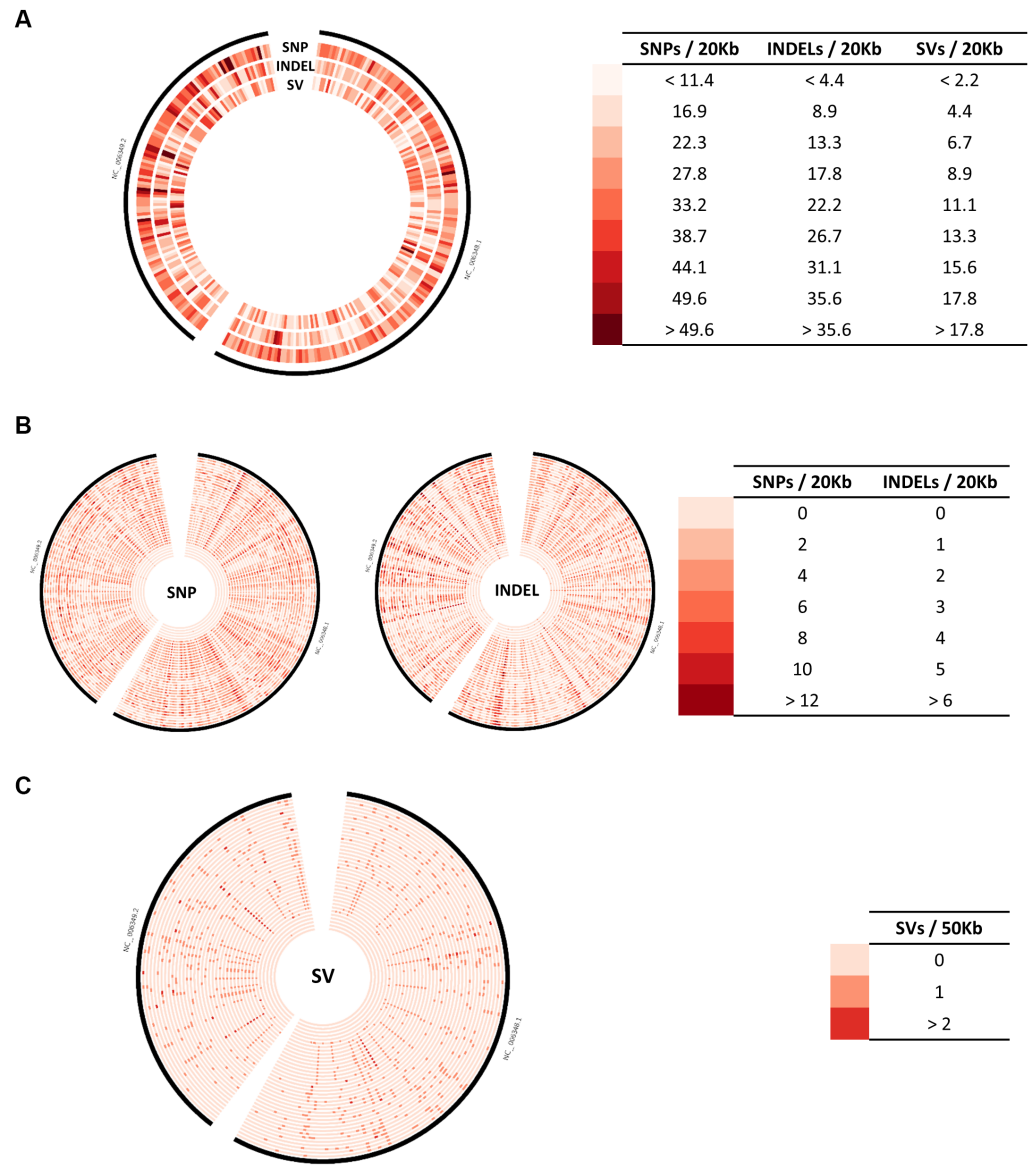
Distribution of genomic variations across the genome of *B. mallei*. **(A)** Distribution of SNPs, INDELs, and SVs across the genome for 112 *B. mallei* strains. **(B)** Distribution of SNPs and INDELS for 38 isolates with complete or chromosome-level genome assembly. **(C)** Distribution of SVs for 38 isolates with complete or chromosome-level genome assembly. The red gradient represents the density of each type of genomic variation.

**Figure 2:**
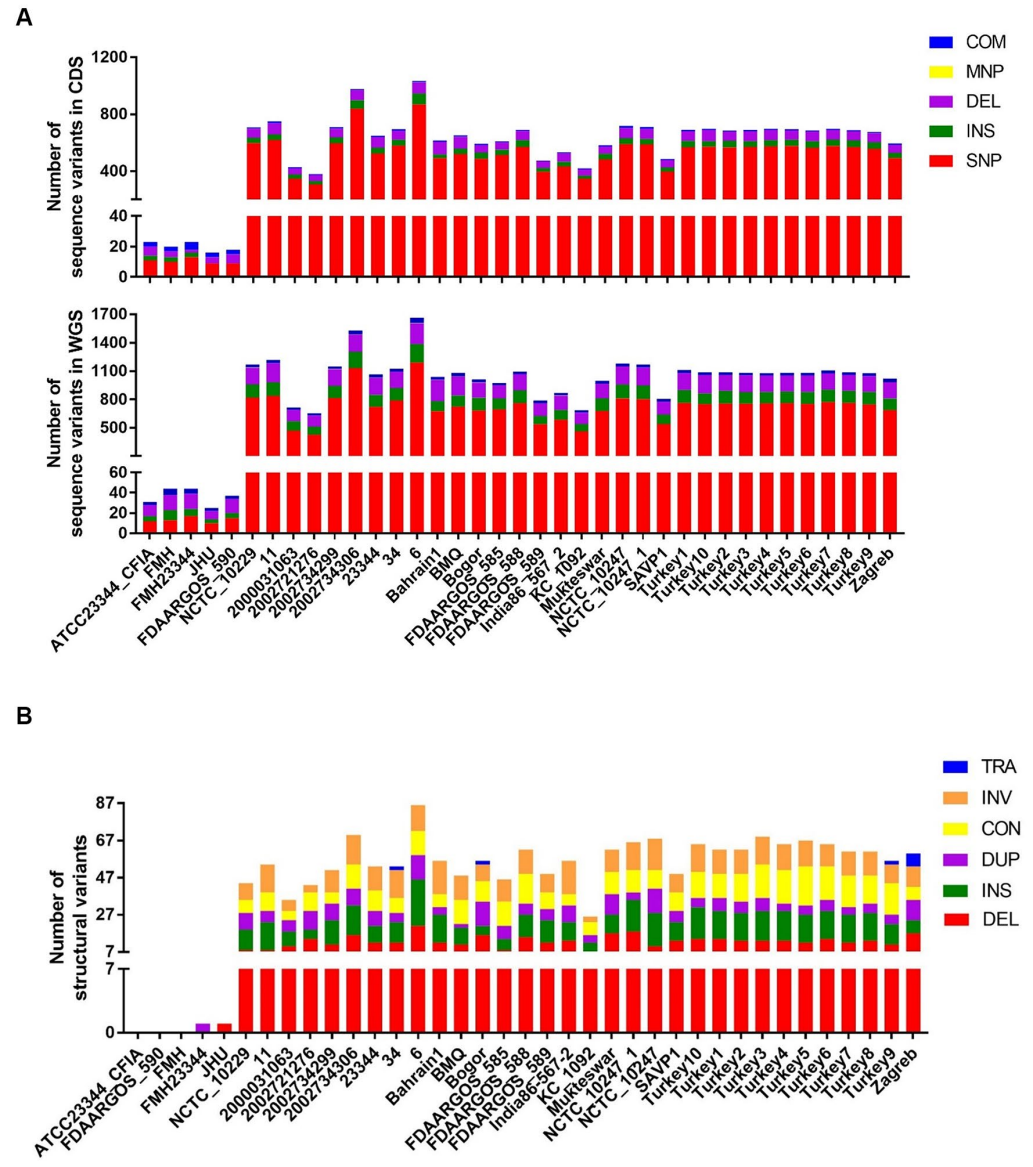
Various types of genomic variations identified in each *B. mallei* strain with complete or chromosome-level genome assembly. **(A)** The number of SNP, INDEL, MNP, and complex variants identified in the coding regions and whole genome sequences. **(B)** The number of multiple types of SVs identified in WGS. SNP, Single Nucleotide Polymorphism; MNP, Multiple Nucleotide Polymorphism; INS, Insertion; DEL, Deletion; COM, Complex (combination of SNP/MNP); TRA, Translocation; INV, Inversion; CON, Contraction; DUP, Duplication.

Due to genetic variability after laboratory or host passages, four *B. mallei* isolates, ATCC 23344, Bogor, Mukteswar, and Zagreb, were re-sequenced using both Nanopore long-read and Illumina short-read sequencing technologies to obtain complete or chromosome-level whole genome assemblies (Supplementary Table S1). In combination with 108 published genomes available on RefSeq (Supplementary File S1), we identified a total of 3,806 core SNPs by using strain ATCC 23344 (RefSeq accession number: GCF_000011705.1) as a reference. The density and distribution of SNPs are similar among chromosome I (CH1) and chromosome II (CH2) (Figure 1A). In addition to SNPs, a large number of INDELs and SVs were identified. Several dense spots of variants indicating common SNPs, INDELs, and SVs are noticeable in multiple sites on both CH1 and CH2 (Figure 1A). To further explore this observation, the distribution and number of SNPs, INDELs, and SVs for 38 strains with complete and chromosome-level genome assembly were examined, respectively (Figures 1B,C). Multiple typical “hotspots” of SNPs and INDELs exist in all genomes, including derivatives of ATCC 23344 as shown in the five inner circles (Figure 1B). Around 70% of small variants, including SNPs, INDELs, MNPs (multiple nucleotide polymorphisms), and complex variants (combination of SNPs and MNPs), were located within coding sequences (CDS) (Figure 2A), some of which introduce early stop codons for several genes, including a gene encoding the serodiagnostic antigen ImpJ (TssK) shown in Table 1. The majority of small variants are still SNPs and INDELs (Figure 2A). Similarly, multiple types of SVs, especially large deletions, in *B. mallei* isolates were identified using strain ATCC 23344 as a reference, except for some ATCC 23344 derivatives (Figures 1C, 2B). These SVs were scattered in both chromosomes, and multiple hotspot regions were observed in most isolates on both chromosomes (Figure 1C).

**Table 1:** Gene-encoded purified proteins used for ELISA development or as potential serodiagnostic markers.

Antigen	Present in ATCC 23344	CH^a^	Gene location number^b^	Locus Tag^c^	Present in *B. mallei* genomes^d^	Present in *B. mallei* genomes (complete or chromosome level)^d^	Present in other non-pathogenic Burkholderia spp^d^	DEG (Zagreb vs. ATCC 23344)	References
FlgK	Yes	1	3143	BMA_RS15840	112/112 (100%)	38/38 (100%)	Y	N	Wagner et al. (49)
Lipoprotein	Yes	1	1320	BMA_RS06655	112/112 (100%)	38/38 (100%)	Y	N	Wagner et al. (49)
Putative exported protein	Yes	1	817	BMA_RS04115	112/112 (100%)	38/38 (100%)	Y	N	Wagner et al. (49)
Peroxiredoxin	Yes	1	1386	BMA_RS06985	112/112 (100%)	38/38 (100%)	Y	N	Wagner et al. (49)
DUF2059 domain-containing protein	Yes	1	407	BMA_RS02025	112/112 (100%)	38/38 (100%)	Y	N	Wagner et al. (49)
OmpA	Yes	1	409	BMA_RS02035	112/112 (100%)	38/38 (100%)	Y	N	Wagner et al. (49)
GroEL	Yes	1	1873	BMA_RS09440	112/112 (100%)	38/38 (100%)	Y	N	Wagner et al. (49)
GroES_1	Yes	1	1874	BMA_RS09445	112/112 (100%)	38/38 (100%)	Y	N	Wagner et al. (49)
O-antigen acetylase	Yes	1	1856	BMA_RS09350	110/112 (98.2%)	37/38 (97.4%)	Y	N	Stone et al. (9)
Flagellin	Yes	1	2696	BMA_RS13600	112/112 (100%)	38/38 (100%)	Y	N	Wagner et al. (49)
GroES_2	Yes	1	2279	BMA_RS11465	112/112 (100%)	38/38 (100%)	Y	N	Wagner et al. (49)
ImpJ (TssK)^e^	Yes	2	3623	BMA_RS26750	78/112 (69.6%)	23/38 (60.5%)	Y	N	Wagner et al. (49)
BopC	No	–	–	–	75/112 (67.0%)	24/38 (63.2%)	Y	N/A	Wagner et al. (49)
BopE	Yes	2	4613	BMA_RS23210	107/112 (95.5%)	35/38 (92.1%)	Y	N	Wagner et al. (49)
BipB	Yes	2	4620	BMA_RS23245	108/112 (96.4%)	36/38 (94.7%)	Y	N	Wagner et al. (49)
Malate dehydrogenase	Yes	2	4835	BMA_RS24325	108/112 (96.4%)	38/38 (100%)	Y	N	Wagner et al. (49)
ABC transporter permease subunit	Yes	2	3525	BMA_RS17765	103/112 (92.0%)	38/38 (100%)	Y	Y	Wagner et al. (49)
Hcp1	Yes	2	3889	BMA_RS19605	105/112 (93.8%)	36/38 (94.7%)	Y	N	Elschner et al. (10)
N-acetylmuramoyl-L-alanine amidase	Yes	2	3447	BMA_RS17365	111/112 (99.1%)	38/38 (100%)	Y	N	Wagner et al. (49)
TssB	Yes	2	3890	BMA_RS19610	105/112 (93.8%)	36/38 (94.7%)	Y	N	Elschner et al. (10)
Phage integrase family protein (0376TH)	Yes	2	4167	BMA_RS21000	111/112 (99.1%)	38/38 (100%)	Y	N	Pal et al. (50)
Hypothetical protein (0375H)	Yes	2	4168	BMA_RS21005	111/112 (99.1%)	38/38 (100%)	Y	N	Pal et al. (50)
BimA	Yes	2	3896	BMA_RS19640	83/112 (74.1%)	36/38 (94.7%)	Y	N	Kumar et al. (51)
TssM	Yes	2	3875	BMA_RS26795	45/112 (40.2%)	11/38 (28.9%)	Y	N	Shanks et al. (52)
TssA	Yes	2	3894	BMA_RS19630	103/112 (92.0%)	35/38 (92.1%)	Y	N	Elschner et al. (10)
Hypothetical protein (A03050H)	No	–	–	–	47/112 (42.0%)	21/38 (55.3%)	Y	N/A	Pal et al. (50)

a Chromosome of ATCC 23344.

b Gene location number match with x axis label of Figure 4B.

c Locus tag of genome assembly of ATCC 23344.

d Using cutoff values: coverage > 80% and identity > 80%.

e Pseudogene with early stop codon in *B. mallei* genomes when compared to *B. pseudomallei* protein.

### Variation in *Burkholderia mallei* gene content (presence/absence of genes)

**Figure 3:**
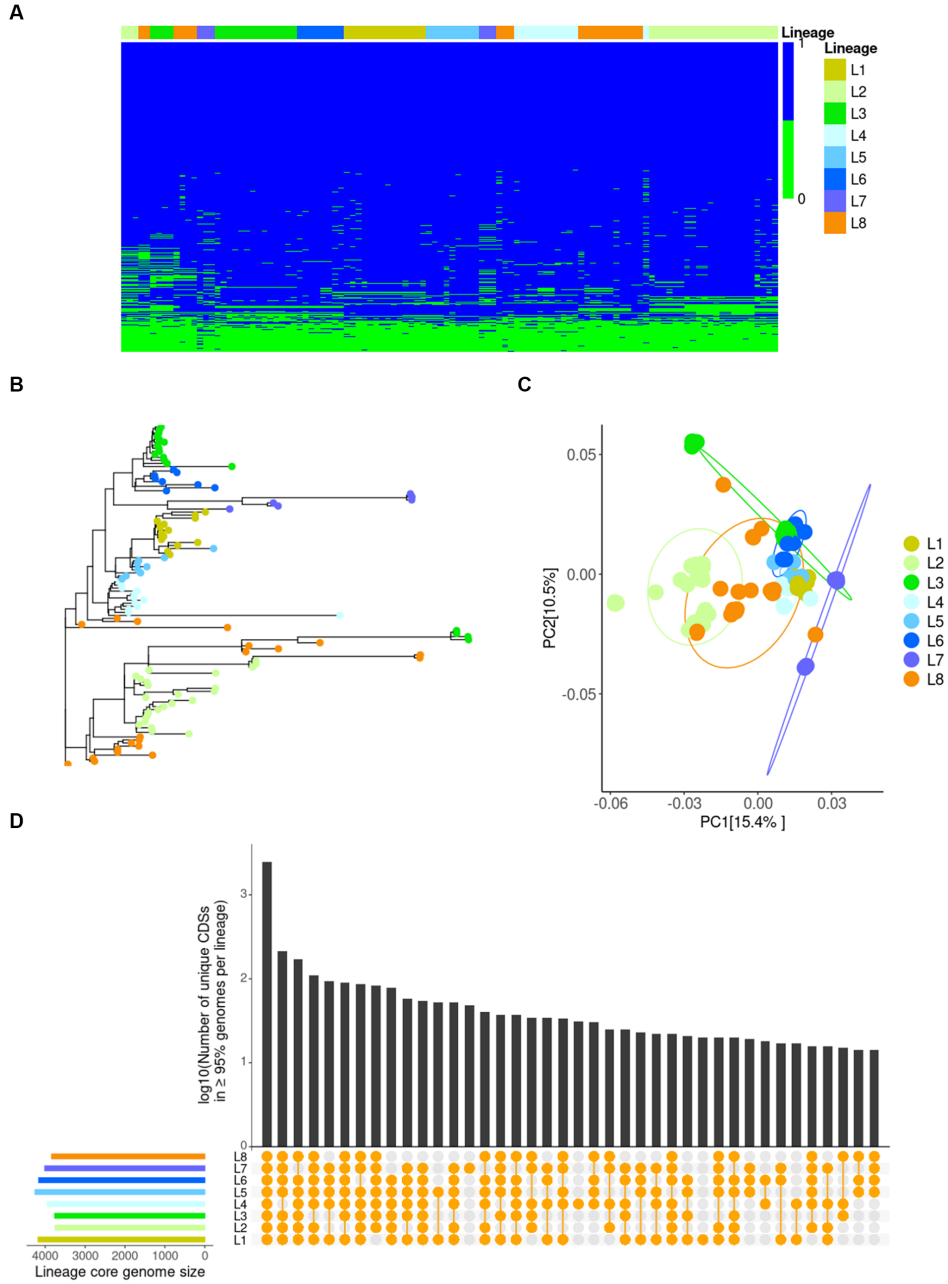
Variations in gene content of *B. mallei* isolates as identified by Pan-genome analysis. **(A)** A presence/absence matrix of 5,332 genes in the *B. mallei* pan-genome. Each row represents a gene cluster. Each column corresponds to a strain’s gene content. The color bar at the top represents different lineages. **(B)** Phylogenetic tree of 112 *B. mallei* isolates using the binary data of presence/absence gene generated by IQtree. **(C)** Principal coordinates analysis (PCoA) plot of gene content between each lineage based on Bray–Curtis dissimilarity. The color represents different lineages. **(D)** UpSetR plot showing the large intersection of lineage-specific core genomes. Intersections between lineages as shown by the presence of a yellow dot in the presence/absence matrix underneath the bar plot. The bar plot represents a logarithm of the number of genes in each intersection.

All 112 available genome assemblies of *B. mallei* were used to identify the core and accessory genomes. Upon successive addition of each genome, the number of gene families in the pan-genome increased to 5,332 and seemed to reach a plateau with a very low γ-value (0.03) of a Heaps’ law fitting, which reflects that *B. mallei*’s pan-genome is nearly closed (38) (Supplementary Figures S1A,B). Overall, 5,332 gene families were identified (Supplementary Figure S1B), in which there were 3,464 persistent genes present in over 95% of the *B. mallei* genomes, 1,386 shell genes that were present at intermediate frequencies, and 482 cloud genes that were found in one, or very few individuals. The gene frequencies tend to show a strongly asymmetric U-shaped distribution with a larger proportion of persistent genes (Supplementary Figure S1B), indicating a lower level of gene gain achieved through horizontal gene transfer (HGT) within *B. mallei* (53). The pan-genome matrix shown in Figure 3A demonstrated substantial variations of gene content among all the genomes of *B. mallei* available in this study. These data indicate gene loss is a significant consequence of *B. mallei*’s genetic variation as limited gene gain was observed.

Gene content variations of a bacterial pathogen have been demonstrated to contribute to various acute and adaptive immune responses elicited by different strains of the same bacterial species (20). Due to the highly plastic genome of *B. mallei* (14), it is highly possible that long term evolution of this species may magnify this phenomenon thereby increasing the complexity of glanders serodiagnosis. To achieve greater accuracy in analyzing gene content variations and avoid unreliable or unavailable metadata mentioned previously (54), we implemented hierarchical Bayesian clustering via FastBaps, which further divided the worldwide phylogeny based on core SNPs into strain-level clusters (Supplementary Figure S2). In total, we identified eight clusters corresponding to different lineages (L1-8) (Supplementary Figure S2). L1 was built up from genomes of the type strain ATCC 23344 and derivatives of ATCC 23344 (US as geographic origin). Other Chinese isolates, such as strain China 5 and ATCC 10399, were found in L5. Interestingly, strains FDAARGOS_587, 2000031063, and KC_1092 were also clustered in L5, an observation which is consistent with previous publications that have pointed out inaccurate metadata for these isolates (15, 54, 55). The majority of isolates from Turkey were grouped into L2. Within the Indian strains in L3, the Budapest strain from Hungary was surprisingly close to strain SAVP1, as previously reported (15). Five genome sequences of possible Russian isolates with SCPM Identifier comprised L4. An exception was the strain SCPM-O-B-7093, which was most closely related to the strains Bahrain1, Bogor, Zagreb, and Mukteswar isolated in the 1990s or 2010s within L6. The genomes of UK strains 2002734306, FDAARGOS_586, and NCTC 120 were grouped with the Turkey strain 6 in L7. The L8 group consisted of strains isolated from different geographical locations and hosts.

Comparing gene contents between different lineages, isolates from the same lineage or close geographic location tended to have rather similar gene contents (Figures 3A,B), except for those of L3 and L8 which overlapped with other lineages. To investigate this finding further, using binary data of the presence/absence of genes, we performed Principal Coordinates Analysis (PCoA) to explore the dissimilarity of gene content between lineages and further confirmed the distinct gene presence-absence variations within L3 and L8 (Figure 3C). Additionally, there were clear differences in gene content between L2 or L7 and the rest of the lineages (Figure 3C), demonstrating that the gene content of the Turkey or UK strains is quite different from those of strains isolated from other geographic locations. Variations in each lineage-core genome (genes present in over 95 percent of the genomes in each lineage) were evident (Figure 3D). All these data demonstrate that *B. mallei* strains from different lineages or isolated from various geographic locations have different gene contents, suggesting various adaptive immune responses can possibly be elicited by strains from different lineage or geographic locations.

**Figure 4:**
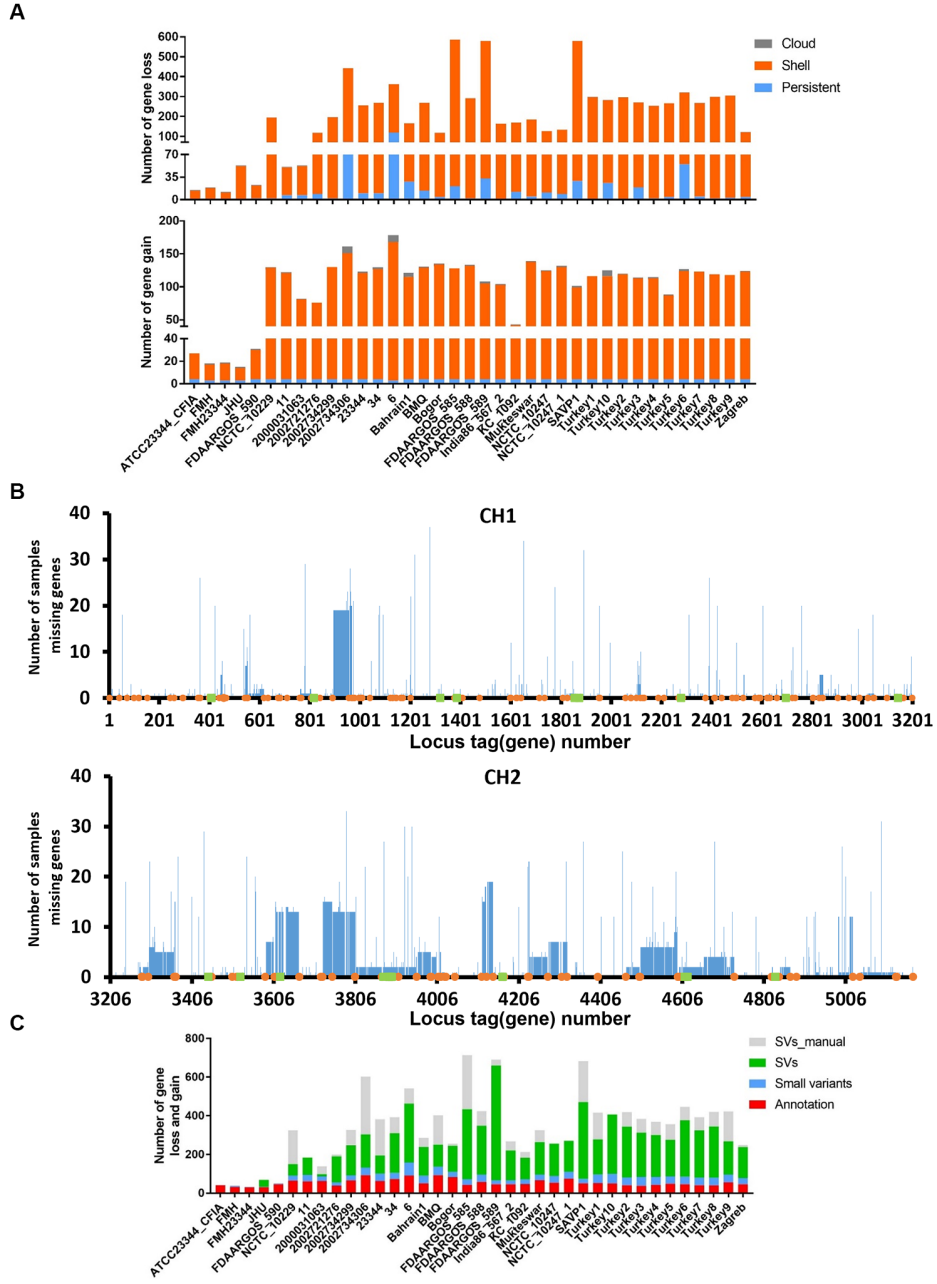
Effects of genomic variations on gene content. **(A)** Number of genes lost and gained (loss in strain ATCC 23344) in each *B. mallei* strain with complete or chromosome-level genome assembly. **(B)** Distribution of gene losses in *B. mallei* strains with complete or chromosome-level genome assembly on the genome of ATCC 23344. The orange dot represents transposase, and the green square shows gene coding antigens shown in Table 1. **(C)** Number of gene losses and gains in each *B. mallei* strain with complete or chromosome level genome assembly, compared to strain ATCC 23344. The color represents the cause of the gene loss or gain. Annotation: genes were not predicted by PGAP; Small variants: gene losses are due to SNPs, INDELs, MNPs, and complex variants. SVs: gene loss due to SVs. SV_manual: gene loss due to SVs that were not identified by MUM&Co.

Moreover, we explored gene content diversity in the complete and chromosome-level genome assemblies to uncover gene presence-absence variations at the individual strain level using strain ATCC 23344 as a reference. Gene loss for each isolate and missing genes in the ATCC 23344 genome (gene gain in other genomes) were identified (Figure 4A). Interestingly, some strains, such as strains 6, 2002734306, and Turkey 6, even lost many persistent genes, while strain ATCC 23344 also lost four persistent genes (Figure 4A). The absence of genes in each isolate was mapped to the chromosomes of ATCC 23344, and multiple hotspots on CH1 and CH2 were observed, especially on CH2 (Figure 4B). Notably, most of the genes encoding antigens, such as BimA, HCP1, TssA, TssB, TssM, A03050H, and 0376H, used in established or developing serodiagnostic tests for glanders or melioidosis are located within those CH2 hotspots and are thus missing from some *B. mallei* isolates (Figure 4B and Table 1). All these data strongly demonstrate that *B. mallei* genome plasticity affects gene content, including some genes which encode proteins used for glanders serodiagnosis.

### Contribution of genomic variations to gene loss

To determine how genomic variations correlated with gene loss, we focused on the cross-comparison of genetic variation with gene content. Although a large number of small variants have been detected in CDS (Figure 2A), resulting in early stop codons and frameshifts, most of the gene loss events were associated with SVs (Figure 4C), which further confirms the key role of SVs in genome plasticity and its role in the loss of genes encoding antigens related to serodiagnosis such as Hcp1, TssB, BimA, and TssA (Figure 4B and Table 1). Additionally, some gene losses are related to the annotation performed by PGAP, which are mainly labeled as pseudogenes due to frameshifts or genes not being annotated (Figure 4C).

### Effects of sequence variations on gene expression

**Figure 5:**
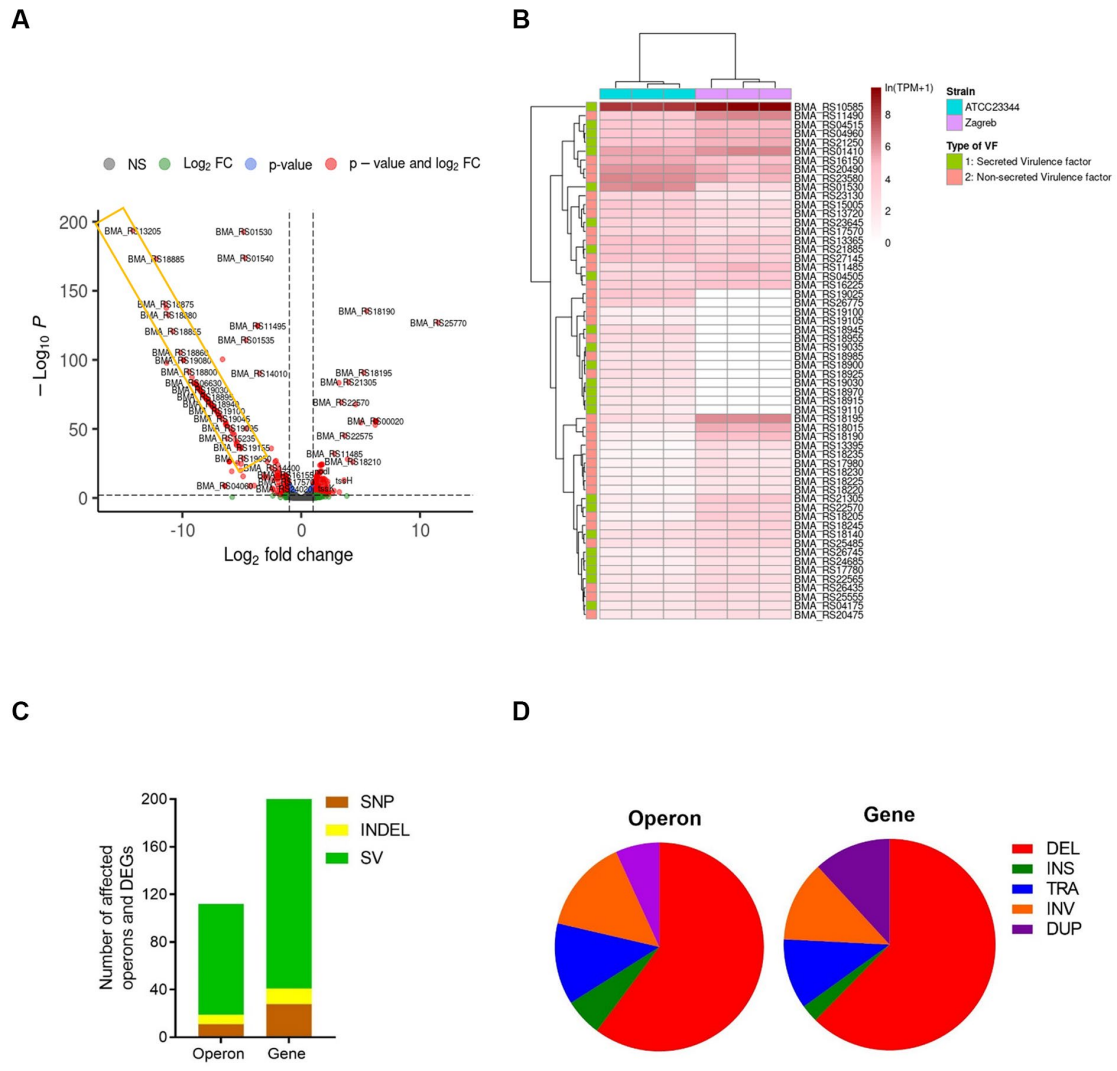
Effects of genomic variants on gene expression. **(A)** Volcano plot to display differentially expressed genes (DEGs) between type strain ATCC 23344 and strain Zagreb of *B. mallei*. The x and y axis represent Log2fold change and –log10P, respectively. The genes indicated in red dots represent the DEGs with |log2 (FC)| > 1 and *p* < 0.001. **(B)** The expression level of 58 differentially expressed virulence genes coding secreted VF (green) and non-secreted VF (pink) between strain ATCC 23344 and Zagreb. The density of the red color represents the gene expression level of ln(TPM + 1). Three biological replicates for each strain. **(C)** The number of SNP (brown), INDEL (yellow), and SV (green) related to affected operons and DEGs. **(D)** The percentage of different SVs related to affected operons and DEGs. The association of genomic variants on DEGs or affected operons was predicted using the simple predictive model that the destruction of the structure of operon and alteration of the non-coding regions, can interfere with gene expression. INS, Insertion; DEL, Deletion; TRA, Translocation; INV, Inversion; DUP, Duplication.

To understand potential effects of sequence variations on gene expression, we compared the transcriptome profiles of strain Zagreb with ATCC 23344 and found slight, but not significant differences overall (PERMANOVA: R2 = 0.415 Pr(> F) = 0.1) (Supplementary Figure S3A), due to a variation of gene expression of less than 10% (Figure 5A and Supplementary File S2). To be specific, only 388 DEGs were identified, for which a total of 155 available KEGG Orthology (KO) were retrieved from the KEGG mapper reconstruction server. The results showed that a total of 82 pathways were affected. The key differences in affected gene functions involve various bacterial metabolic pathways, pathogenesis, and virulence, including ABC transporters, quorum sensing (QS), two-component system, and biofilm formation (Supplementary Figure S3B). Specifically, the expression of ABC transporter permease subunit, which has been regarded as a potential serodiagnostic marker (56) was significantly different between these two strains (Table 1). Since proteins related to bacterial pathogenesis and virulence are typically used as serodiagnostic biomarkers, we investigated expression differences for virulence factors. A total of 842 virulence factors and 2,294 potential virulence factors were predicted using the PathoFact pipeline (Supplementary File S3). Fifty-eight (58) out of the 842 virulence genes, which code for 24 secreted and 34 non-secreted virulence factors, were among the identified DEGs, including some genes related to the type IV secretion system (Figure 5B and Table 2).

**Table 2:** DEGs associated with SVs.

Locus_Tag	Gene	Product	Operon	SV type
BMA_RS10585		Hypothetical protein	OPERON_1002	Translocation
BMA_RS18190	*tssC*	Type VI secretion system contractile sheath large subunit	OPERON_1710	Translocation
BMA_RS18195		Type VI secretion system tube protein Hcp	OPERON_1710	Translocation
BMA_RS18205	*tssF*	Type VI secretion system baseplate subunit TssF	OPERON_1710	Translocation
BMA_RS18900		Hypothetical protein	OPERON_1783	Deletion
BMA_RS18915		Porin	OPERON_1786	Deletion
BMA_RS18925		LuxR C-terminal-related transcriptional regulator	OPERON_1788	Deletion
BMA_RS18945		Hypothetical protein	OPERON_1790	Deletion
BMA_RS18955		LysR family transcriptional regulator	OPERON_1791	Deletion
BMA_RS18970		Porin	OPERON_1793	Deletion
BMA_RS18985		PucR family transcriptional regulator	OPERON_1794	Deletion
BMA_RS19025		Hypothetical protein	OPERON_1798	Deletion
BMA_RS19030		Cytochrome c peroxidase	OPERON_1799	Deletion
BMA_RS19035		Alkaline phosphatase family protein	OPERON_1799	Deletion
BMA_RS19105	*mhpT*	3-(3-hydroxy-phenyl)propionate transporter MhpT	OPERON_1809	Deletion
BMA_RS19110		Porin	OPERON_1810	Deletion
BMA_RS22565		Efflux transporter outer membrane subunit	OPERON_2149	Deletion
BMA_RS26775		Hypothetical protein	OPERON_1804	Deletion

In an attempt to understand the effects of genomic variants on gene expression, we focused on not only changes in transcription related to the alteration of operon structures mediated by SVs, but also changes of non-coding regions upstream of operons, including promoter and regulator regions, mediated by SNPs or INDELs, respectively. Using transcriptomic data of strain ATCC 23344, OperonSEQer predicted a total of 2,455 operons (Supplementary Files S4, S5). One hundred and eighty-five of the DEGs were located within 108 operons disturbed by at least one type of genomic variant, especially SVs, with a large number being caused by deletions (Figures 5C,D). A large deletion from locus_tag BMA_RS18805 to BMS_RS19170 was observed in the genome of strain Zagreb, which was also reflected in transcriptomic data shown within the yellow rectangle in Figure 5A. Besides deletions, translocations also had a big impact on the expression of many genes, including several virulence genes (Figure 5D and Table 2).

## Discussion

Glanders diagnosis mainly relies on serological tests. Apart from different assay platforms, selection of appropriate antigens used in serological assays is critical to achieving adequate diagnostic sensitivity and specificity. Due to its simple standardization and objective analysis, ELISAs using recombinant proteins as antigens have shown very promising results with a potential replacement of CFT for glanders serodiagnosis in the near future (6). However, in the current study, we showed that, due to its high plasticity, the genome of *B. mallei* contains large-scale variants, resulting in differences in gene content and expression among each isolate. A number of gene-encoded proteins, including antigens used for the development of glanders ELISAs and virulence factors, are deleted, modified, or inactivated, in some isolates thereby compromising the efficacy of serodiagnostic tests using purified recombinant antigens.

Using all the *B. mallei* genome sequences available online and in-house, we undertook a comprehensive investigation of the nature of genetic variations of *B. mallei* isolates. SNPs are the most common type of genetic variation. The rate of single nucleotide alterations generated upon passage or infection has been reported as being typically very low (17). This suggests that SNPs may have a minor effect on genome plasticity, which is further supported by the small number of core SNPs identified in our study. Therefore, SNPs may provide a good level of discrimination and avoid genome alteration during passages. Indeed, the whole genome SNP-based phylogenetic analysis presented in this study demonstrates a high resolution of relationships among *B. mallei* isolates collected from diverse geographical locations (Supplementary Figure S2). Our phylogeny is quite similar to the phylogenetic relationships obtained from previous cgMLST (15, 54) or WGS-based SNP typing analyses (57). Compared to the previous three lineages (57) or 12 clusters (BHR, UAE, IND1/2/3, CHN1/2, TUR1/2/3, HUN, RUS) (15), hierarchical Bayesian clustering (FastBaps) used in this study subdivided the phylogeny into eight lineages (L1-8). The previously defined L1 is the same as our L7 (57), containing strain 6 and 20027344306 (NCTC120 or FDAARGOS_586), while previously reported L2 was further subdivided into current L1 (cluster CHN2), L5 (cluster CHN1), L3 (cluster IND1), and L6 (clusters IND2 and BHR). The previously defined L3 consisted of our L2 (cluster TUR1), L4 (cluster RUS) and L8 (clusters TUR 2, TUR3, HUN, and IND3).

Furthermore, our eight lineages match clusters identified through high-resolution melting PCR (PCR-HRM) (58). Except for L8 containing assemblies from three subtypes: L3B2, L3B3sB1, and L3B3sB3, each lineage is comprised of only genomes from the same PCR-HRM cluster, such as L1 (L2B2sB1Grp1), L2 (L3B3sB2), L3 (L2B2sB2), L4 (L3B1), L5 (L2B2sB1Grp2), L6 (L2B1), and L7 (L1). It is likely that L8 can be further subdivided using FastBap, which may match PCR-HRM cluster analysis. The lineage analysis in this study will provide new clues to enhance future molecular typing.

Besides SNPs, a relatively small number of INDELs were also identified across all *B. mallei* genomes available in RefSeq (Figure 1B). In contrast to consequences of SNPs, it is believed that there are some links between the accumulation of INDELs and the evolution of *B. mallei* (17). INDELs in both coding and non-coding regions were observed in the *B. mallei* genome upon passage *in vitro* and *in vivo*, thereby resulting in a few ORF frameshifts and alteration of gene expression, respectively, (17). Similar results can be seen in our data (Figures 2A, 4C, 5C).

It is generally believed that *B. mallei* evolved from an ancestral *B. pseudomallei* following serendipitous infection of a living host followed by ongoing evolution in which the genome underwent large-scale chromosome rearrangements and reduction (16, 17). Following this change, *B. mallei* continues to evolve with additional large genome reductions resulting from chromosomal rearrangements, which represent a significant contributor to genome plasticity (16, 17). Therefore, it is not surprising that a large number of SVs, specifically deletions, were observed in many *B. mallei* isolates (Figures 1C, 2B). So far, consequences, causes, and underlying mechanisms of SVs in *B. mallei* have not been well characterized. Our analysis revealed that SVs, in particular large deletions, are a significant cause for gene loss (Figure 4C) and altered operons, resulting in interference with structural gene expression (Figure 5D). Additionally, the strain JHU, obtained from a laboratory-acquired infection of *B. mallei* ATCC 23344, had evidence of an approximately 97 kb deletion starting from 1,226,811 to 1,324,421 on the CH2, compared to the reference strain ATCC 23344, which had not been reported in the previous study (17), thus suggesting that chromosome rearrangement could also occur in *B. mallei* during infection.

Consistent with this study, a previous report clearly indicated that the smaller secondary chromosome evolves more rapidly than the larger one, showing greater substitution rates and gene dispensability (59). This secondary chromosome usually serves as an evolutionary testing ground where genes are weakly preserved (18), which may explain the higher frequency of gene loss on the *B. mallei* CH2, especially *in vivo* (17). Based on previous transcriptional data, transposases on CH1 were down-regulated, and most of the over-expressed genes were located on CH2 *in vivo* (60), Over-expression of transposases on CH2 may be associated with instability of CH2 *in vivo*, which can be investigated and confirmed by future studies.

Genetically different strains of the same pathogenic bacterial species can elicit markedly diverse host immune responses during infection (20). The presence of large scale genetic changes in *B. mallei* may amplify this phenomenon during long–term evolution, particularly *in vivo,* as demonstrated in other *Burkholderia* species (61–64). Many genes in *B. mallei* CH2 have virulence or virulence-related functions, playing a pivotal role in pathogenesis (60). The extensive gene losses observed in CH2 (Figure 4B) could potentially lead to a reduction in its virulence during infection, thereby contributing to *B. mallei’s* ability to transmit and colonize between hosts. This serves as an example of optimized interaction between pathogenic bacteria and hosts to maximize their reproductive success, such as transmission, survival, and colonization (65). However, due to gene loss and alteration of gene expression, it is highly possible that antigens may be disrupted at the genomic or transcriptomic levels during infection, a phenomenon demonstrated in previous studies. For example, one spleen isolate of *B. mallei* obtained post-infection displayed a change in LPS phenotype, from smooth to rough, due to loss of O-polysaccharide during infection (66). Additionally, several *B. mallei* strains, such as *B. mallei* ATCC 23344 human and horse isolates, showed several gene deletions, including BMAA0367 (GNAT family N-acetyltransferase), BMAA0623, BMA2996, BMAA0729 (TssM), and BMA0685 (TonB) due to INDELs (17). TssM and TonB are well-known virulence factors of *B. mallei* (52, 67). Furthermore, TssM in *B. pseudomallei* has been investigated as a potential serodiagnostic biomarker (68) and used to develop a vaccine for melioidosis (69). BMAA0367, a GNAT family N-acetyltransferase, has not been well characterized in *Burkholderia* spp., but it was up-regulated during *B. mallei* infections (60), indicating that it is associated with *B. mallei*’s pathogenesis. Here, we found that genes encoding several antigens used to develop ELISA or potential serodiagnostic biomarkers, such as TssB, TssA, BimA, A03050H, and Hcp1, are not present in several *B. mallei* complete genome assemblies, due to genomic variations, but are present in other environmental *Burkholderia* spp. (Table 1). Most importantly, most of these genes are located within hotspots of gene loss on CH2 (Figure 4B). Loss of these gene-encoded antigens or virulence factors in *B. mallei* may be more likely to occur during natural infection compared to laboratory culturing since genome evolution in bacterial pathogens can occur faster and at higher frequency during the span of short host infections (13, 70–72). Based on the instability of CH2, it would be optimal to identify stable, unique, and conserved serodiagnostic biomarkers using genes located on CH1, such as GroEL (73). Moreover, by targeting multiple conserved serodiagnostic antigens from different lineages in a single serodiagnostic test, we could mitigate the effects of specific antigen loss and could cover different lineages, leading to a potential improvement in sensitivity and specificity.

Genomic variations, especially structural variations, have a significant impact on overall gene expression. Unfortunately, most SV studies mainly focus on humans and other multicellular eukaryotic organisms. To study the effects of multiple types of variants on gene expression in bacteria, we focused on the *B. mallei* strain Zagreb. This strain was chosen due to its wide usage for diagnostic antigen production and the presence of a large number of inter-chromosomal translocations observed in its genome when compared to ATCC 23344 (data not shown). Using the simple predictive model that the destruction of the operon structure and alteration of the 5′ flanking non-coding regions, including promoters and transcriptional regulators, can interfere with gene expression, this study has shown that genomic variations, specifically SVs, can alter gene expression, including some virulence genes (Figure 5B and Table 2). Interestingly, deletion of LuxI/LuxR-type QS systems observed in the strain Zagreb may also be indirectly altering the expression of many other genes, including those of virulence factors, toxins, and biofilm components (74).

In this study, we used all available *B. mallei* genome assemblies in the RefSeq database and assemblies of four additional in-house *B. mallei* strains. It is important to note that the quality of bacterial genome assemblies in the RefSeq database may vary. To address this issue, we assessed the quality of the 112 genome assemblies and annotations using BUSCO’s completeness score (Supplementary File S1) for quality control. However, it should be acknowledged that the presence of sequencing and assembly errors, in addition to accuracy of aligners and variant callers, may affect the genetic variation identification performed in our study (75). Nevertheless, this limitation must be taken into consideration. Besides that, bacterial gene expression and regulation are complicated and have yet to be fully elucidated, so it is hard to comprehensively investigate the effect of genomic variations on gene expression using the limited sample size in this study. Furthermore, there is a significant difference in gene expression profiling of a *B. mallei* isolate between *in vivo* and *in vitro*, particularly with most of the over-expressed genes on CH2 *in vivo* (60). This indicates *in vitro* transcriptional data may not be instrumental in predicting *in vivo* gene expression and regulation, and the possibility that virulence genes or serodiagnostic markers located on CH2 show variable expression between different isolates during infection will be investigated further. However, it is likely that the disruption of operon structures by SVs, such as deletion and translocation, impacts the expression of genes within or near the operon during infection as well, since these co-regulated genes under a common control system are interrupted (46). The specific impacts of SVs on gene expression needs to be further investigated using more transcriptome data, especially in relation to bacterial infection and virulence factors.

## Conclusion

In summary, we have demonstrated that the plasticity of the *B. mallei* genome interferes with its gene content and expression. Accordingly, methods of glanders serodiagnosis using only purified recombinant antigens could be potentially compromised. Multiple instances have demonstrated that certain targeted antigens, including BimA, HCP1, TssA, TssB, TssM, A03050H, or potentially valuable antigens like virulence factors, may undergo loss or significant changes in expression due to genetic variations. As a result, these alterations can potentially lead to modifications in the immune response, including antibody production, during infection. This study provides novel insights into *B. mallei* genome plasticity, including details and consequences of genomic variation. These findings will be useful for informing improved glanders serodiagnosis and molecular typing in the future.

## Data availability statement

The whole-genome assemblies of strains ATCC 23344, Zagreb, Mukteswar and Bogor have been deposited in GenBank under accession number CP124607-CP124608, CP124605-CP124606, CP124602-CP124603, and CP124604, respectively. All the raw sequencing data are available under BioProject number PRJNA954830. Specifically, the RNA-sequencing raw data are available in the NCBI Sequence Read Archive under accession numbers SRR24153017 to SRR24153022 and whole genome sequencing raw data are available in the NCBI Sequence Read Archive under accession numbers SRR24860065 to SRR24860072.

## Author contributions

MK designed the experiments. MK, RG, and PC wrote the manuscript. PC, RG, JC, EH, SN-D, DC, JH, KM, M-OD, and MK reviewed and edited the manuscript. JC, EH, and SN-D performed WGS. PC, MK, RG, JC, and M-OD analyzed sequencing data. DC, JH, and KM conducted bacterial culture and RNA purification experiments. All authors contributed to the article and approved the submitted version.

## Footnotes

^1^https://github.com/AstrobioMike/bit#bioinformatics-tools-bit

^2^https://astrobiomike.github.io/unix/ncbi_eutils

^3^https://github.com/rrwick/Porechop

^4^https://github.com/rrwick/Filtlong

^5^https://github.com/nanoporetech/medaka

^6^https://github.com/tseemann/snippy

^7^https://github.com/SAMtoBAM/MUMandCo

^8^https://github.com/iqtree/iqtree2

^9^https://github.com/gtonkinhill/fastbaps

^10^https://itol.embl.de/

^11^https://github.com/labgem/PPanGGOLiN

^12^https://cran.r-project.org/web/packages/ggplot2/index.html

^13^https://cran.r-project.org/web/packages/pheatmap/

^14^https://github.com/vegandevs/vegan

^15^https://github.com/vegandevs/vegan

^16^https://www.kegg.jp/kegg/mapper/reconstruct.html

## Supplementary material

SUPPLEMENTARY FIGURE S1 Pan-genome analysis of 112 *B. mallei* genomes. (A) Rarefaction curves for the core (persistent) genome size and pan-genome. (B) U-shaped distribution demonstrating gene cluster count among 112 *B. mallei* genomes.

SUPPLEMENTARY FIGURE S2 SNP-based Phylogenetic tree of *B. mallei* isolates and related metadata. The phylogenetic tree was obtained by IQtree with 1000 ultrafast bootstraps. Lineages (L1-8) were determined by Fastbap. Geographic location was collected from BioSample and Assembly database from NCBI: Bahrain (BH), Brazil (BR), China (CN), Hungary (HU), India (IN), Indonesia (ID), Iran (IR), Myanmar (MM), Pakistan (PK), Russia (RU), Turkey (TR), United Kingdom (UK), United States (US), and Yugoslavia (YU).

SUPPLEMENTARY FIGURE S3 Differences in gene expression and Kyoto Encyclopedia of Genes and Genomes (KEGG) pathways between strain ATCC 23344 and Zagreb. (A) PCoA plot of gene expression between strain ATCC 23344 and Zagreb based on Bray-Curtis dissimilarity. Three biological replicates for each strain. (B) KEGG pathways correlating to virulence DEGs between strain ATCC 23344 and Zagreb. The red color bars represent pathways related to bacterial pathogeny and virulence.

SUPPLEMENTARY Table S1 Summary of genome assembly information.

SUPPLEMENTARY Table S2 Number of reads for each sample in RNA-seq experiment.

SUPPLEMENTARY FILE S1 Genomes and assemblies used in this study.

SUPPLEMENTARY FILE S2 Summary of DEGs generated from RNA-seq analysis.

SUPPLEMENTARY FILE S3 Virulence factors predicted by PathoFact.

SUPPLEMENTARY FILE S4 Operons on CH1 predicted by OperonSEQer.

SUPPLEMENTARY FILE S5 Operons on CH2 predicted by OperonSEQer.
