# Supplementary figures and images for: Influence of genomic variations on glanders serodiagnostic antigens using integrative genomic and transcriptomic approaches
Source: Front Vet Sci. 2023 Dec 6;10:1217135. doi: 10.3389/fvets.2023.1217135 (PMC10730941; doi:10.3389/fvets.2023.1217135)

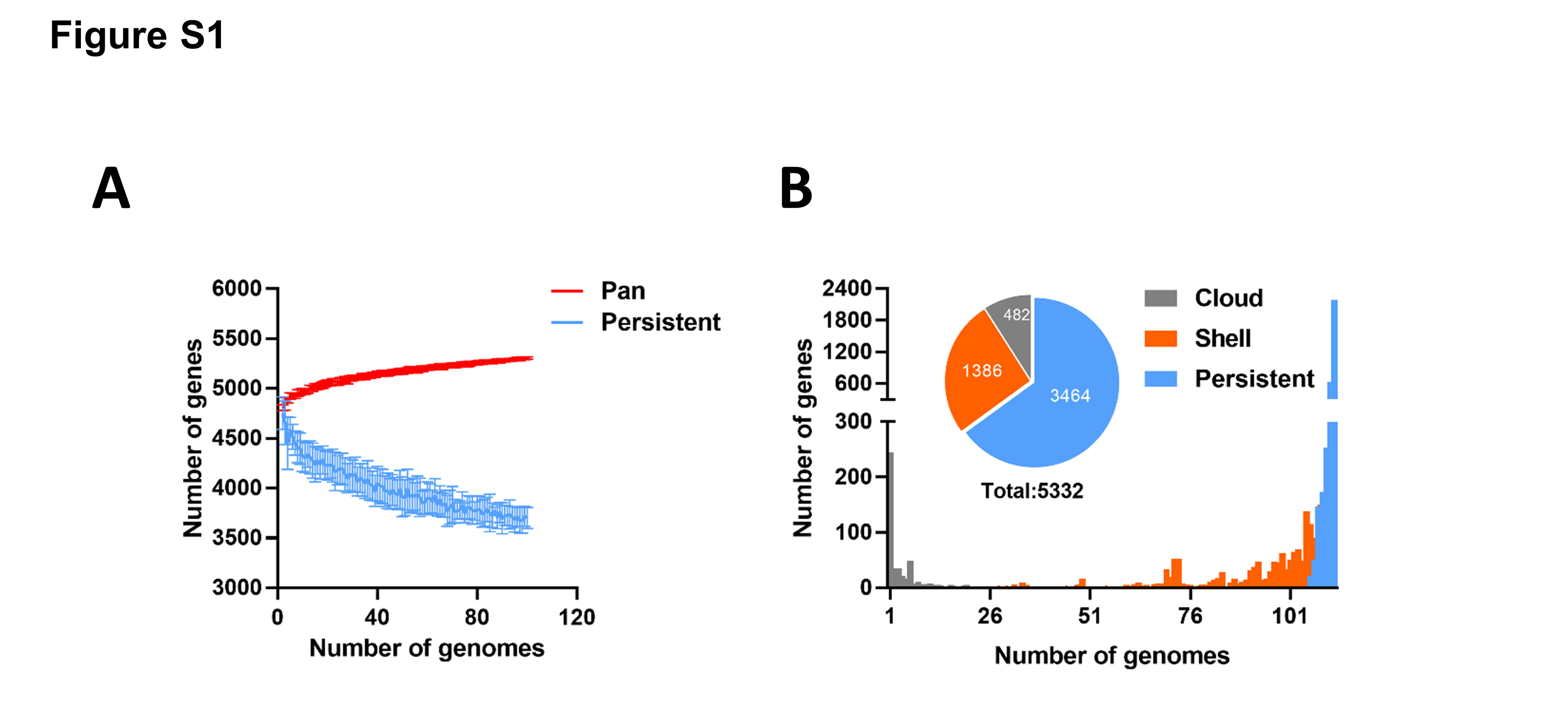

Supplement: SUPPLEMENTARY FIGURE S1 — Pan-genome analysis of 112 B. mallei genomes. (A) Rarefaction curves for the core (persistent) genome size and pan-genome. (B) U-shaped distribution demonstrating gene cluster count among 112 B. mallei genomes. [file Image_1.TIF]

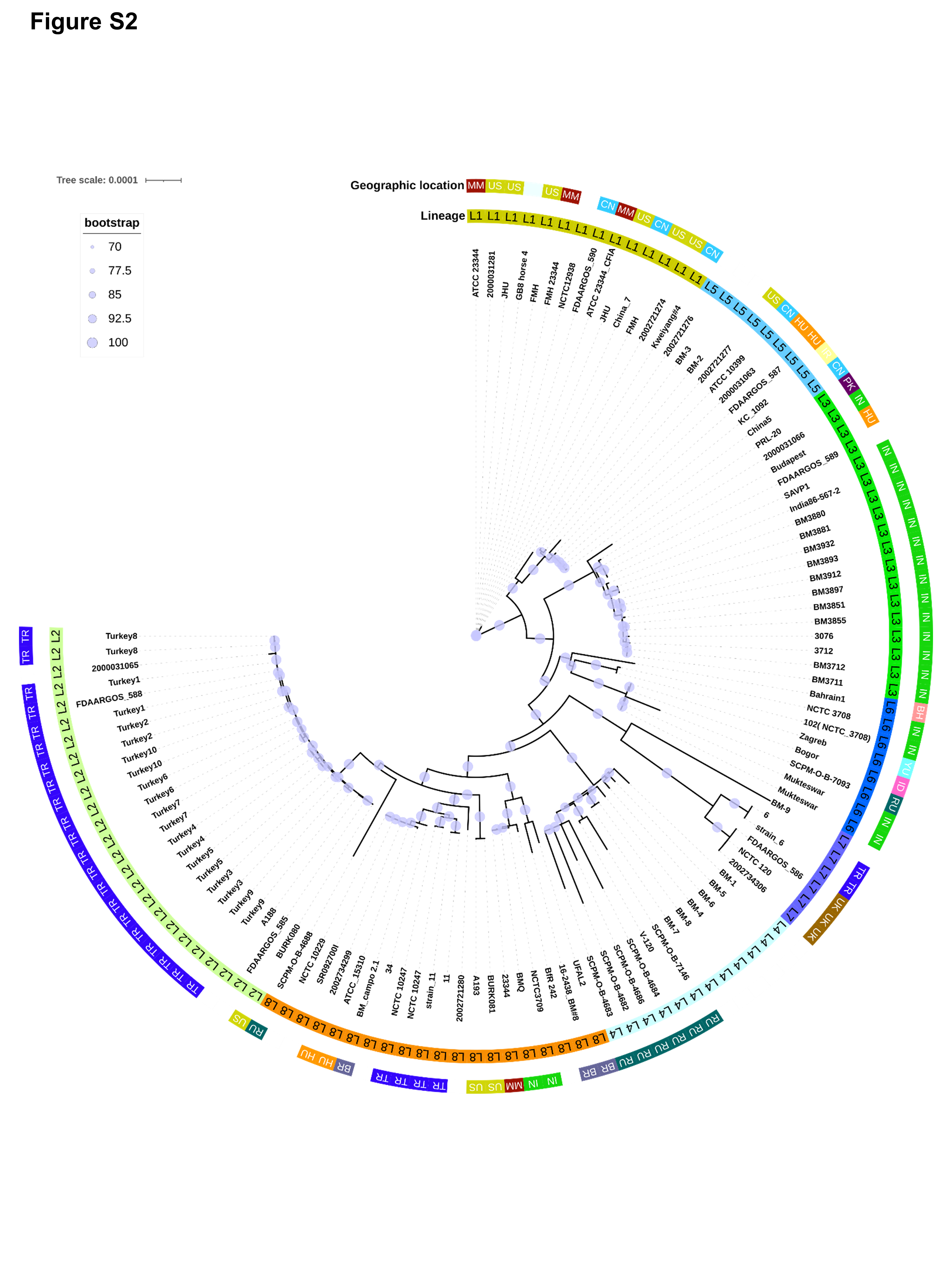

Supplement: SUPPLEMENTARY FIGURE S2 — SNP-based Phylogenetic tree of B. mallei isolates and related metadata. The phylogenetic tree was obtained by IQtree with 1000 ultrafast bootstraps. Lineages (L1-8) were determined by Fastbap. Geographic location was collected from BioSample and Assembly database from NCBI: Bahrain (BH), Brazil (BR), China (CN), Hungary (HU), India (IN), Indonesia (ID), Iran (IR), Myanmar (MM), Pakistan (PK), Russia (RU), Turkey (TR), United Kingdom (UK), United States (US), and Yugoslavia (YU). [file Image_2.TIF]

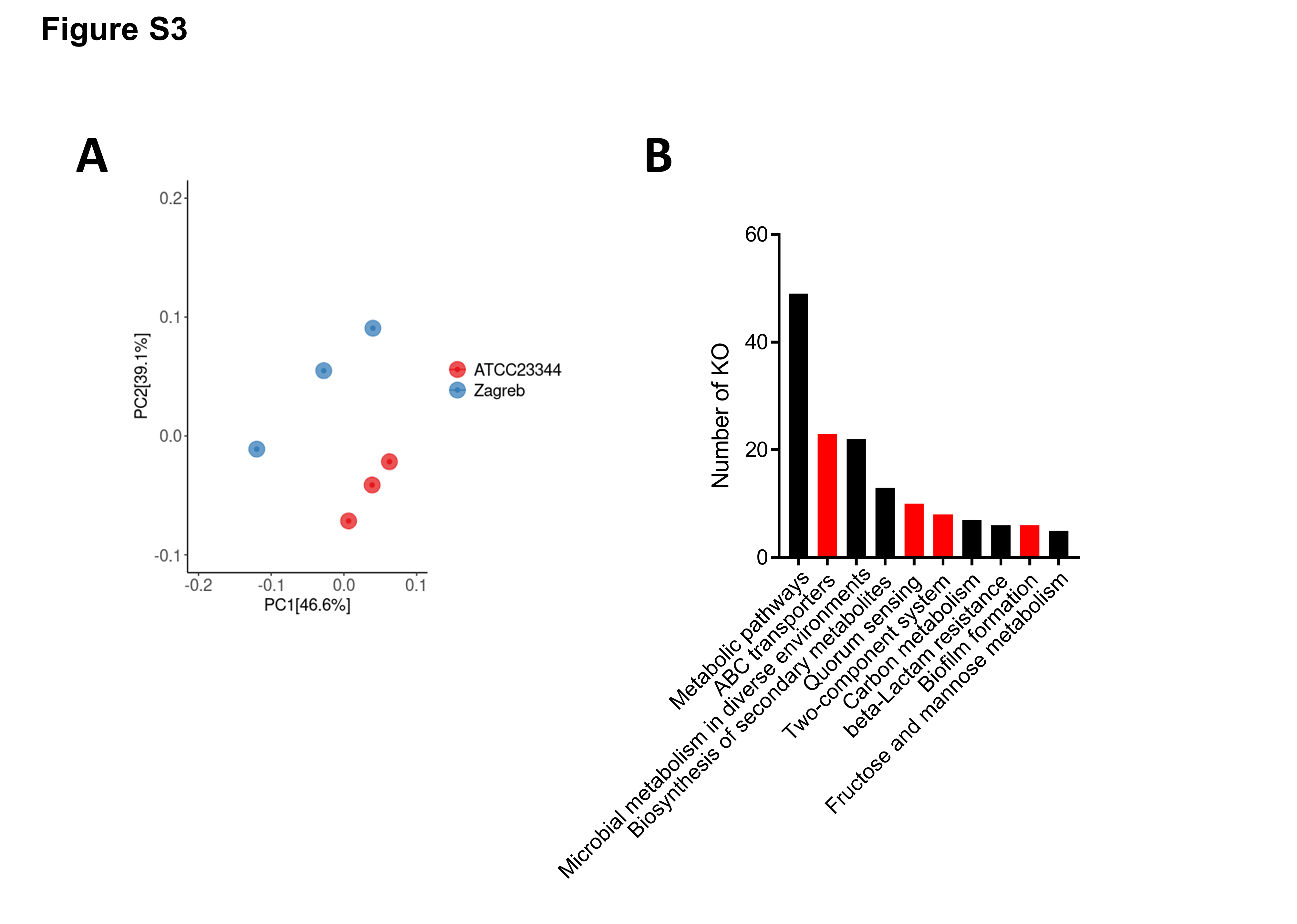

Supplement: SUPPLEMENTARY FIGURE S3 — Differences in gene expression and Kyoto Encyclopedia of Genes and Genomes (KEGG) pathways between strain ATCC 23344 and Zagreb. (A) PCoA plot of gene expression between strain ATCC 23344 and Zagreb based on Bray-Curtis dissimilarity. Three biological replicates for each strain. (B) KEGG pathways correlating to virulence DEGs between strain ATCC 23344 and Zagreb. The red color bars represent pathways related to bacterial pathogeny and virulence. [file Image_3.TIF]
