# Supplementary material for: Influence of genomic variations on glanders serodiagnostic antigens using integrative genomic and transcriptomic approaches
Source: Front Vet Sci. 2023 Dec 6;10:1217135. doi: 10.3389/fvets.2023.1217135 (PMC10730941; doi:10.3389/fvets.2023.1217135)
Supplement: SUPPLEMENTARY Table S1 — Summary of genome assembly information. [file Table_1.DOCX]

**Table S1. Summary of genome assembly information.**

|  | Nanopore | | | | Illumina | |
| --- | --- | --- | --- | --- | --- | --- |
| Strain | # Reads | Mean Length  (bp) | N50 (bp) | Coverage | # Reads | Coverage |
| ATCC 23344 | 98,541 | 9,133 | 11,661 | 151X | 2.4M | 110X |
| Bogor | 115,393 | 8,963 | 9,279 | 172X | 2.2M | 103X |
| Mukteswar | 112,917 | 9,035 | 9,313 | 175X | 3.0M | 148X |
| Zagreb | 40,304 | 14,391 | 15,592 | 100X | 3.2M | 150X |
