# Supplementary material for: Influence of genomic variations on glanders serodiagnostic antigens using integrative genomic and transcriptomic approaches
Source: Front Vet Sci. 2023 Dec 6;10:1217135. doi: 10.3389/fvets.2023.1217135 (PMC10730941; doi:10.3389/fvets.2023.1217135)
Supplement: SUPPLEMENTARY Table S2 — Number of reads for each sample in RNA-seq experiment. [file Table_2.DOCX]

**Table S2. Number of reads for each sample in RNA-seq experiment**

| Sample | Number of Reads |
| --- | --- |
| ATCC23344-1 | 18,507,934 |
| ATCC23344-2 | 24,063,144 |
| ATCC23344-3 | 20,110,264 |
| Zagreb-1 | 38,834,872 |
| Zagreb-2 | 17,000,090 |
| Zagreb-3 | 21,481,210 |
